# Optimism, ambivalence, and opportunities: staff perspectives signaling a critical turn in patient-oriented research in forensic mental health care settings

**DOI:** 10.3389/fpsyt.2026.1810931

**Published:** 2026-05-18

**Authors:** Christopher Canning, Greg Procknow

**Affiliations:** 1 Waypoint Research Institute, Waypoint Centre for Mental Health Care, Penetanguishene, ON, Canada; 2 Department of Psychiatry, University of Toronto, Toronto, ON, Canada; 3 Critical Disability Studies, York University, North York, ON, Canada

**Keywords:** critical disability studies, descriptive qualitative analysis, epistemology, forensic mental health, mad studies, patient-oriented research

## Abstract

This qualitative descriptive study examines the views of 25 clinicians, clinical leaders, and senior hospital leaders about forensic patient-oriented research (POR) practices and the inclusion of patients as research partners in forensic mental health care environments. Data from semi-structured interviews were analyzed inductively using thematic analysis to pinpoint predominant themes. Perspectives on forensic POR materialized across three attitudinal domains: optimism, ambivalence, and opportunities. Findings underscore positive overtures toward including patient voices in research and consensual support for forensic POR, despite obvious structural and organizational barriers, power inequities, risks of tokenism, and epistemic concerns inherent in forensic settings. Many agreed that this ‘shiny new object’ is worth exploring, despite still being an emerging approach in secure environments. We discuss how the findings align with current literature about staff perspectives on participatory research and more critical approaches in Critical Disability and Mad Studies and conclude with recommendations for future research.

## 1 Introduction

Forensic mental health care systems vary considerably across jurisdictions but share a common mandate to provide treatment and rehabilitation for individuals with serious mental illness who have come into contact with the criminal justice system, typically because they are being assessed for or have been found to be Not Criminally Responsible (NCR) or unfit to stand trial (UST) (1). In systems with adequate resources, multidisciplinary teams of psychiatrists, psychologists, nurses, social workers, occupational therapists, and other allied staff deliver care across a spectrum of security levels, typically characterized by strict safety measures including secure perimeters, locked units, surveillance systems, and closely supervised movement (1, 2). In Canada, patients remain under the jurisdiction of provincial or territorial administrative tribunals, which provide regular oversight and issue dispositions to the most appropriate secure treatment setting until such time as the person is deemed to no longer pose a threat to public safety. While forensic hospitals provide access to outdoor, recreational, and vocational programming, patients continue to experience these environments as restrictive (2), with stays determined by ongoing risk assessments rather than fixed time periods, which creates the potential for long and indeterminate hospitalizations (3, 4).

A central challenge of forensic mental health care globally is the dual mandate of promoting recovery and reintegration while ensuring the safety of patients, staff, and the public. Forensic services are slowly orienting towards more patient-centeredness and recovery-oriented approaches in accord with democratization ideals (5, 6). As Livingston describes, patient-centered care in forensic settings is “an approach to providing health care in a manner that emphasizes and respects the needs, values, and choices of patients” (5). Patient-centered approaches respect patients’ agential capacities by prioritizing treatment needs and recovery over their index offence (7). Access to services and community programming varies across forensic settings and is shaped by clinical and organizational factors, with multidisciplinary teams monitoring recovery benchmarks until Review Boards adjudge that a patient is well enough and does not pose a risk to public safety to step down or return to the community under conditional or absolute discharge (7, 8). These conditions shape the context within which forensic patient-oriented research must operate.

This study examines the perspectives of 25 clinicians, clinical leaders, and senior hospital leaders about forensic patient-oriented research (POR) practices and the inclusion of patients as research partners in forensic environments. We respond to a gap in the literature, which has not yet investigated healthcare provider and hospital leader perspectives on POR and related participatory research approaches in forensic mental health care settings. Forensic POR applies patient-centered principles, such as power sharing, authentic engagement, and dignity in secure forensic settings, where momentum is growing to advance such projects (8–10). Nevertheless, research on POR in these environments remains limited (11).

In 2011, the Canadian Institutes of Health Research (CIHR) launched the national Strategy for Patient-Oriented Research (SPOR) (12). SPOR aims to “integrate patients as active partners in health research that will lead to improved health outcomes and an enhanced health care system” (13, 14). This approach aims to provide patients with decisional power and translate co-produced research into practice, policy, and health system improvements. POR also seeks to build research capacity and involvement among healthcare providers, healthcare administrators, researchers, and consumers/users from study design to implementation (12).

Published research capturing the attitudes of non-researcher mental health clinicians and healthcare administrators toward involving patients in mental health research remains limited (15, 16). Several reflective articles document favourable responses among non-service-user hospital staff in secure settings, including perceptions of partnering with lived experience researchers and service users as insightful and an opportunity to reciprocate and share power (17); evidence of shifts in power differentials (18); interest in making research “more relevant to both service users and mental health practice more generally” (19); identification of factors important for cultivating collaborative service-user research in secure inpatient settings (20); thoughts on coproducing research with patient partners (21); attitudes held by staff toward coproduction in secure mental health care (22); and the shared experiences of co-produced research between clinicians and patients (18, 23).

Other studies signal resistance to patient engagement (24) and highlight perceived barriers, such as patient voices shifting the research agenda away from its originally intended purpose (14), or how clinicians view their research priorities aligning with those service users propose (25). However, no studies that we found have explicitly addressed the value service providers and hospital leaders attach to including forensic patients in participatory research projects, or systematically solicited healthcare provider perspectives on POR in forensic mental health research generally, despite early calls by Faulkner and Morris, who asked professionals, “What do you think user involvement would add to the research process?” (19).

## 2 Methods

This paper was guided by the following research questions: *How do forensic healthcare providers and hospital leaders view participatory research, particularly forensic patient-oriented research? How do forensic healthcare providers and hospital leaders view patient voices in research? How do perspectives differ, or not, between frontline providers and clinical and administrative leaders*?

### 2.1 Study design

We chose a qualitative descriptive methodology because it is well-suited for exploring the perspectives of healthcare leaders within patient-centered health care organizations (26). We adopted a descriptive qualitative design to examine the perspectival phenomena of healthcare providers’ and hospital leaders’ views on forensic POR and the integration of patient voices in research. This approach is an established methodological tradition that has been successfully used to advance participatory action research in forensic mental health settings (27, 28). Qualitative description is particularly appropriate when researchers seek a straightforward account of the informational content of the data (29), which is best articulated in participants’ own phrasing and word choices (30).

Importantly, description is never void of interpretation; researchers’ accounts of phenomena inherently involve interpretive processes (31). Likewise, participants selectively share events, situations, or experiences, and in doing so, they engage in thoughtful interpretation of their experiences before recounting them (31). Descriptive designs offer flexibility and enables researchers to stay close to the data while choosing techniques that best align with their aims (32), without committing to any predetermined theoretical framework or lens (33).

### 2.2 Data collection

This study was situated at a mental health hospital in Canada, which has secure forensic mental health units that serve only men, alongside other specialized inpatient and outpatient mental health programs that serve women and men. Recruitment entailed promoting the project in the hospital newsletter, posting paper flyers in all inpatient forensic units, and emailing clinical staff and leaders. All eligible staff received email messages to their professional inboxes with a link to the study’s information sheet and consent form. Using maximum variation (heterogeneity) sampling to ensure a breadth of perspectives was represented, 31 hospital staff were interviewed as part of a larger project exploring barriers and facilitators to POR in forensic mental health care settings (9, 34, 35). Scheduling interviews proceeded confirmation of consent. Data were collected through semistructured in-person or videoconferencing interviews between November 2023 and January 2024. Interviews ranged from 45 to 60 minutes and were recorded and transcribed verbatim by a third-party professional service using human transcriptionists. Respondents received a $30 electronic gift card upon completion of their interview. See Appendix A for a list of interview questions.

### 2.3 Data analysis

The full dataset of 31 interviews was collected as part of a larger study examining barriers and facilitators to forensic POR (9). For this paper, we conducted a sub-analysis of 25 interviews and re-coded the data using an inductive thematic approach, which deploys pre-set research questions to narrow the scope of a study (36) to address the three guiding research questions. Six interviews did not meet the inclusion criteria. The goal of this analysis was to isolate and better understand how perspectives differ across frontline clinicians, clinical leaders, and senior hospital leaders, given that each group occupies distinct organizational roles, responsibilities, and proximities to patients, and therefore may support, resist, and/or operationalize POR in unique ways in forensic mental health care settings. We did not use a fixed or planned theory or framework to analyze the interviews.

Coding generally followed Braun and Clarke’s (37) six recursive phases, performed manually in Microsoft Word by G.P., who was recruited after the interviews were conducted, and C.C, the project PI. The interviews were conducted by C.C. and two former project team members. G.P. first familiarized himself with the full data corpus (37) and documented initial reflections in a log, then conducted open coding of meaningful text segments to generate 62 codes using Word’s comment feature. Codes were grouped into categories, reviewed collaboratively by C.C. and G.P. for theme alignment, and refined into 16 subthemes across three higher-order themes (optimism, ambivalence, opportunities); we excluded five non-contributory subthemes after testing for research question relevance. Themes were narratively defined to capture their ‘essence,’ supported by anonymized excerpts. Together, G.P. and C.C. provided an analytical narrative for each theme, identifying the story each theme told in relation to the guiding research questions (37). G.P. drafted the manuscript and C.C. reviewed and edited the draft. All responses in the Findings below have been deidentified and anonymized to prevent recognition.

G.P. and C.C. are both lived experience researchers who carry their positionality into the interpretation and discussion of the descriptive findings, while striving to keep the coding process grounded in participants’ accounts. G.P. is a consumer of psychiatric services, twice-diagnosed with schizophrenia, but has settled on schizoaffective disorder – a bipolar disorder and schizophrenia cross-venture he likens to a confused chimera, combining multiple disorders. He has found an intellectual and disciplinary base in Critical Disability Studies and Mad Studies. G.P. approaches forensic POR through a critical psychiatry lens. C.C. is a sociologist and identifies as a consumer of mental health services, both personally and as a family caregiver to a late parent with lifelong and disabling depression. He approaches his research and leadership through a pluralist epistemological position and ambivalent activist lens, which acknowledges the space for “seemingly contradictory feelings, thoughts, and attitudes towards madness and related interventions and systems” while fighting against systemic injustices that are “real and deeply felt” (38). Our reflexive process involved discussions about the philosophical and methodological considerations of POR, highlighting tensions between reformist and more critical views about forensic mental health systems. We discussed how critical theory should guide forensic mental health research but rarely does, as well as the tensions between idealist and pragmatic approaches to meaningful patient involvement in restrictive settings. These discussions informed our interpretive decisions around themes and subthemes, including how we understood the disparity between leaders’ rhetorical enthusiasm and frontline staff’s more pragmatic reservations.

### 2.4 Participants

We sorted participants into three categories to avoid identifying them among a relatively small data set: clinicians (e.g., nurses, forensic care assistants, occupational therapists, recreation therapists), clinical leaders (e.g., clinical managers and directors), and senior hospital leaders (e.g., senior administrators). To protect confidentiality and anonymity, each participant was assigned an alphabetical identifier rather than a professional title or unit designation. Each participant was also assigned an abbreviated role label (CL for clinicians; CLL for clinical leaders; and SHL for senior hospital leaders) paired with their alphabetical identifier to help readers follow participant quotes and excerpts (e.g., A, CLL). These identifiers denote individuals only and do not correspond to specific rank or clinical/service area. See **Table 1** for a list of participants and role labels.

**Table 1:** Participant table.

Clinicians (CL)	Clinical leaders (CLL)	Senior hospital leaders (SHL)
O	A	B
P	F	C
Q	G	D
R	N	E
S		H
T		I
U		J
V		K
W		L
X		M
Y		

## 3 Findings

In this section, we present findings from 25 interviews with frontline staff, clinical leaders, and senior hospital leaders, organized into three overarching themes: optimism, ambivalence, and opportunities (see **Table 2** for a list of all themes and subthemes). Each theme captures a distinct orientation towards forensic POR and illustrates perspectives from hospital staff and leaders across various departments, positions, and levels, including ethical and justice-based considerations to hesitations about safety to pragmatic and relational considerations.

**Table 2:** Themes and sub-themes.

Theme	Subtheme
Optimism	Ethical, justice-based rationale and recognition of patient knowledge
	Learning with and from patients to improve care, outcomes, and systems
	Culture change: challenging stigma, paternalism, and custodial models
	Patients know what to research
Ambivalence	Structural and organizational constraints
	Engagement, motivation, and recruitment uncertainties
	Epistemic worries and what counts as knowledge
	Power, tokenism, and the politics of patient centred labels
Opportunities	Markers of success
	Gaining new skills, keeping busy, and personal benefits
	Shiny new object

### 3.1 Theme 1: optimism

This theme captures participants’ expressions of genuine enthusiasm and perceived benefits of forensic POR, including ethical, relational, and cultural outcomes that can benefit staff and patients.

Overall, clinical and senior hospital leaders were the most optimistic about and supportive of the idea of forensic POR, with a smaller but clear subset of frontline clinical staff being more cautious, critical, and, in a few instances, stigmatizing towards patients and patient knowledge. The supportive responses from leaders were longer, richer, and more elaborate than critical comments. All nine senior leaders clearly expressed enthusiasm or saw advantages of forensic POR. Across these leaders, there are well over a dozen separate supportive passages, often several per interview, describing forensic POR as “long overdue” (B, SHL), “transformative” (M, SHL), “mutually beneficial” (C, SHL), and “possibly more valuable” (A, CLL) than other research paradigms. Other senior leaders describe forensic POR as an “exciting evolution” (I, SHL), a social justice-oriented approach (M, SHL), and a powerful way to “get more in the heads of patients” to understand their experiential realities (J, SHL).

Frontline clinical staff accented general approval for forensic POR, suggesting that it is “a wonderful opportunity” (R, CL), “quite imperative” (Y, CL), and “bold … really, really cool and different” (S, CL). Frontline staff, more than leaders, emphasized personal and measurable benefits this research approach could confer on patients, with two indicators being “confidence” (R, CL) and “fulfillment” (W, CL). Below we describe this optimism in more detail, captured across four subthemes.

#### 3.1.1 Subtheme 1.1: ethical, justice-based rationale and recognition of patient knowledge

Frontline staff and hospital leaders framed forensic POR as ethically the right thing to do and grounded in social justice, respect, and a duty to recognize forensic patients’ experiential knowledge as legitimate and overdue in research. Forensic POR is described as something patients are owed in a system that has historically silenced or marginalized them, especially given their “captive” (C, SHL) status in secure mental health care environments. Leaders more often articulated this rights- and justice-based language explicitly. For example, one senior leader suggested that forensic patients “have a right to have a say in terms of … what research we conduct and the implications of that research. In an ideal world, I think the advantage of [forensic POR] is social change” (M, SHL). Another senior hospital leader echoed this statement: “I think we absolutely need to include [patients] in research, particularly when it impacts their … care and environment, if we’re going to make progressive and steady change” (L, SHL). Another clinical leader spoke to the importance of hearing from patients directly as experts of their own experiences:

[T]his type of research gives the patients an opportunity to have their voice heard and documented. It can make someone feel empowered to know that their words are actually going to be shared with other professionals across the field, possibly internationally. Because … so often people’s voices are not heard … I think ultimately it will help us create better services to hear from patients directly because [they are] experts in mental health, far more than we are, and experts in the forensic system. (A, CLL).

#### 3.1.2 Subtheme 1.2: learning from patients to improve care, outcomes, and systems

Forensic POR was described as a way of accessing a “treasure trove” of experiential knowledge and moving away from top-down control (J, SHL). Participants emphasized that engaging with patient perspectives can surface and challenge what is “already known” (J, SHL), encouraging researchers and healthcare providers to think differently about improving care (G, CLL) and potentially health outcomes (C, SHL; L, SHL). One senior leader noted that “bringing outcomes to life” could help others understand why forensic POR is valuable (J, SHL). Frontline clinical staff similarly expressed that when patients help to shape questions and interpretations of research, the resulting work becomes more relevant (Q, CL; W, CL). A hospital leader noted that sitting with patients and truly hearing from them can challenge bias and pre-held assumptions, leading to concrete changes in practice, including providers’ professional behaviour and policy (M, SHL). As one clinical leader reflected, listening to patient voices contributed to their own professional growth: “I have learned way more from direct clinical experiences and hearing from the people we serve” (A, CLL).

Frontline staff responses generally highlighted optimism about how forensic POR processes and outcomes could improve conditions at the patient or unit levels and could generate operational changes within their remit of influence. However, they were more critical of strategic top-down mission shifts they felt neglected their safety and concerns, whereas leaders focused on the merits of forensic POR to overhaul culture or organizational-specific transformation.

#### 3.1.3 Subtheme 1.3: culture change: challenging stigma, paternalism, and custodial models

Throughout the interviews, leaders linked forensic POR to a broader culture shift away from custodial, risk-dominated models toward therapeutic and relational-oriented care and research. One senior hospital leader noted how POR is part of the cultural shift currently underway at the hospital:

[POR] is an important part of our journey in the way that we’re shifting culture from more custodial to a more … therapeutic approach. And … those issues are wider and reflect … the way that patients are seen or not seen as people with experiences and perspectives that [they are] able to share, and that are important for us to consider not only in care, but also in research. (K, SHL).

Furthermore, involving patients more authentically in research, we heard, is expected to challenge stigma about forensic patients’ capabilities, counter paternalistic assumptions about who can generate knowledge (J, SHL; M, SHL) and normalize patient involvement in decision-making. Paternalism was only noted by senior leaders (J, SHL; M, SHL). Forensic POR was perceived as providing staff glimpses into “some of the biases and … [our] paternalistic ways” presently rooted in psychiatric systems of care (J, SHL).

Clinical and senior leaders also acknowledged that persistent stigma about the value of forensic patients’ contributions exists in these settings and hypothesized that forensic POR could reduce mental health stigma (B, SHL; C, SHL; H, SHL; M, SHL). Leaders used the label ‘stigma’ in the interviews; frontline staff never did. Because frontline staff tended to focus on more immediate, patient-level issues, they were more likely than leaders to place themselves in patients’ shoes when considering factors that might affect forensic POR. For example, they highlighted contextual barriers to POR, including “liberty restrictions” (V, CL), experiences of seclusion or restraint (X, CL), and a sense of being treated like “zoo animals” to be studied by unfamiliar people (S, CL), including researchers who were strangers to the patient (W, CL).

#### 3.1.4 Subtheme 1.4: patients know what to research

Frontline staff and leaders noted that when patients help decide what to study and how to study it, research has the potential to be more meaningful to patients (B, SHL; K, SHL). One senior hospital leader observed, for example, that while clinicians often focus on illness outcomes, patients may be more concerned with the side effects of treatment (B, SHL). Leaders remarked that excluding patient voice surfaces the hospital’s broader “areas of failure” (L, SHL), whereas frontline staff were more concerned about that leaders might judge them personally based on the shortcoming that patients might identify. One clinical leader also suggested that patients’ voices might hold greater “clout” if incorporated into forensic POR findings, compared to when concerns are expressed outside of research contexts and coming informally from a patient directly (A, CLL).

Frontline staff expressed strong enthusiasm for forensic POR in terms of how patients can make research more relevant. One frontline clinician was “surprised [forensic POR] has not been done widely,” calling it “exciting” and “illogical” to exclude patients, especially for long-stay patients who can gain skills and a sense of purpose through participation (O, CL). Similarly, another frontline clinician described forensic POR as “the best way to get relevant research” and “a few steps ahead of where we are right now,” implying that it could enhance engagement and perceptions of fairness in hearings and transitions (Q, CL).

Despite broad support for forensic POR, participants simultaneously expressed reservations, sometimes within the very same interviews, revealing a more ambivalent and layered set of perspectives. We detail these below.

### 3.2 Theme 2: ambivalence

This theme captures the obvious tensions between participants’ support for and hesitations about forensic POR in forensic mental health settings. Despite an overwhelming optimism about forensic POR described in Theme 1, frontline staff, clinical leaders, and senior leaders all raised apprehensions and mixed feelings about it.

However, these ambivalent reflections tended to be fewer in number and often embedded within otherwise supportive narratives. For example, one senior leader flagged the fear of risk and a “litigious” environment that may steer people away from “tough questions,” even while strongly endorsing the need for such questions (C, SHL). Two other senior leaders highlighted patient transience and uncertainty about patient willingness to be on research teams as essential issues to address (D, SHL; E, SHL). One clinical leader stressed that organizational barriers, multiple layers of approval, and the difficulty of “getting anything done” in forensic mental health settings put forensic POR at risk (F, CLL).

Frontline staff raised far more concerns than leaders about forensic POR; each frontline clinician offered at least one cautious passage or critical stance. We noticed that most participants framed more critical claims as if they were relaying the views of unnamed colleagues. We wondered whether staff were expressing concerns vicariously using distancing statements such as “they,” “some staff,” or “most staff” to avoid admitting or taking responsibility for their own critiques or concerns. In other words, participants appeared to use other staff as proxies to communicate their reservations toward the culture of forensic environments, and thus the feasibility of forensic POR. For instance, remarks such as, “[S]o I have heard little things like…” (R, CL) or “they feel” (Q, CL) may indicate elements of social desirability bias or impression management, in which staff present themselves to the researcher more favourably by projecting views that challenge the hospital and research culture onto others rather than themselves. These concerns are captured further below.

#### 3.2.1 Subtheme 2.1: structural and organizational constraints

Participants emphasized that forensic environments are bureaucratic, slow to change, and highly structured by security and legal status, which creates obvious challenges for forensic POR. Time constraints, described as requiring a “slow and steady build” (S, CL) or a need for “more proof to ensure buy-in” (J, SHL), along with transience (D, SHL), security demands (D, SHL; J, SHL), and conflicts with clinical duties (“we already are super stressed getting charting done” (Q, CL)) were cited as serious constraints. Short-stay or high acuity units were viewed as challenging contexts for ongoing collaboration, whereas extended-stay, tertiary units with more stable patients were seen as more feasible (D, SHL).

Frontline staff and clinical leaders also pointed to operational pressures such as “lower staff counts” (Q, CL), restrictive practices (W, CL), medication routines, and other tasks “necessary to the human rights of several clients” (F, CLL), all of which leave patients with limited unstructured time. Limited “short windows” for engaging patients further constrain involvement (P, CL).

Senior leaders described progress in forensic POR as slow and “messy” in otherwise fastpaced systems (B, SHL; M, SHL). Others framed the path forward as a “long, upwards sloping road” (K, SHL) requiring patience and acceptance of structural hurdles (F, CLL; J, SHL).

#### 3.2.2 Subtheme 2.2: engagement, motivation, and recruitment uncertainties

Frontline staff and leaders questioned how many patients will want to take on sustained, team-level roles in forensic POR rather than simply participating in one-off studies. They described challenges related to insight, competing priorities, misinterpretations (C, SHL), and mismatched expectations regarding topics (S, CL), as well as additional support patients may need to remain engaged over time. For example, one senior leader wondered “how much willingness” there will be for patients “to be a part of [a team],” especially since many do not want to be in the hospital in the first place (E, SHL).

Several leaders noted that issues of consent and capacity may complicate patient involvement in forensic POR, making recruitment more challenging. They emphasized that consent could fluctuate: a patient may agree to participate on one day and withdraw on another or may be capable of some decisions but not others (E, SHL). A frontline clinician suggested that if a patient is incapable of consenting to treatment, they should likewise be excluded from forensic POR projects (H, SHL). Leaders were primarily concerned with the risk of decompensation among patient partners, which could affect capacity, participation, and the ability to provide informed consent (D, SHL; E, SHL; H, SHL).

Frontline staff, conversely, focused less on fluctuating capacity and more on selecting appropriate participants based on their ability to contribute meaningfully (O, CL; R, CL; S, CL; V, CL; W, CL). Whereas leaders linked capacity changes with risks of patient dropout, frontline staff framed their concerns as practical considerations about matching tasks to patients’ abilities. Leaders also raised concerns about the potential for psychological harm. Several suggested that participation in POR, or exposure to its subject matter, could “trigger” or “retraumatize” patient partners (C, SHL; R, CL; S, CL), underscoring a protective stance centered on vulnerability and clinical risk. Staff, more than leaders, focused on patient-level limitations to participating in research, emphasizing diminished cognition, active psychiatric symptoms (R, CL), and treatment-related side effects (O, CL; P, CL) as issues that could compromise research quality, even introducing “delusional content” (P, CL). Ten responses from six frontline staff proposed developing a tool to screen out patients perceived as insincere or acutely unwell (P, CL; U, CL), and consulting with care teams to assess patient capacity to contribute (S, CL). Frontline staff were particularly concerned about manipulation, stressing the need to assess intentions among potential participants. As one frontline staff noted, “[those] who are going to volunteer … are going to be the ones that are going to take advantage of it … and take advantage of … lesser functioning patients” (V, CL).

#### 3.2.3 Subtheme 2.3: epistemic worries and what counts as knowledge

We recorded epistemic challenges as a form of hesitation, reflecting doubts about interpreting and integrating patient accounts as legitimate forms of knowledge. Some leaders acknowledged missed opportunities arising from the failure to include patient voices, noting, for example, that “it’s easy to think we know best” (L, SHL). Frontline staff in particular described difficulty seeing past patients’ index offences. Others noted a reduced quality of patient contributions when symptoms drive speech content rather than rational intent or judicious thought (O, CL; R, CL). Other participants touched on the probability of patients self-censoring input or contributions, fearing reprisal or judgment from staff (“maybe one of the staff members will roll their eyes when they respond” (R, CL). Some staff expressed concerns about patients’ truthful accounts when well and untruths when unwell (P, CL; X, CL). Staff described the challenges of sifting through the “actual from factual” from delusion (O, CL; R, CL). From our perspective, the assumption that a patient is being manipulative might constitute a form of epistemic injustice, as manipulation is invoked to justify discounting patients’ voices and denying them epistemic equivalency.

Some frontline staff worried about falsities being taken at face value (W, CL) and how false complaints could undermine data quality (P, CL; S, CL). One frontline staff described “the potential for people to not be completely forthcoming” and to present themselves as “complete victims of the system” (T, CL). One provider noted that patients might use forensic POR as an avenue to air grievances and to use it “as points to get out of here” (S, CL).

#### 3.2.4 Subtheme 2.4: power, tokenism, and the politics of “patient-centered” labels

Participants recognized deep power imbalances between staff and patients and worry that, without clear benefit and shared decision-making, forensic POR could become tokenistic. Only leaders used the term ‘tokenistic’ or a variation of it (C, SHL; D, SHL; M, SHL); no frontline staff used this language. Demke et al. define tokenism as “pseudo participation ‘for show’ or to meet formal requirements. It is unethical per se, as it is based on the claim that it will benefit those people personally concerned, although it often mainly benefits the ones who organize participation” (39).

One senior leader explained that they would “almost rather … do less [POR] than do lots of tokenistic patient-oriented work” (M, SHL). A second leader remarked that “the common pitfalls around tokenism [is that] it’s just going to be one person. … You see that done so often, which is painful” (C, SHL). Some staff and leaders were concerned with the extractive nature of POR. If researchers leave having extracted information from both frontline staff and patients, they are taking away and often leave little behind in their absence (M, SHL; P, CL).

Some staff also found the language of “patient-centered research” problematic as a label in itself (V, CL), feeling it sidelines staff perspectives and safety, which may hinder buy-in even when they accept the underlying principles and goals. One clinical leader noted how staff buy-in operates at an organizational level and relationally, shaping what patients hear and whether participation in research might be welcomed:

Staff will be your biggest barrier. If the staff do not believe in [POR], if the staff don’t feel that it is important, then that’s going to be the message that the patients are getting. And I think … on programs where maybe they don’t have the best relationships with their patients … I wonder if the patients will feel like they should be participating in something like this if [they] end up in a more negative relationship with the staff. (N, CLL).

To help mitigate these concerns, one participant suggested alternatives such as “staff and patient safety research” to reduce resentment and increase staff buy-in (V, CL). Some responses signaled that involvement may be superficial if power does not genuinely shift (D, SHL). Frontline staff, however, perceive leaders as resisting patient involvement in forensic POR because it would require administrators to relinquish power (Y, CL). Conversely, one senior leader admitted that “part of it may also be a bit protectionist in terms of … exposing our own words” (J, SHL). These comments highlight some discomfort among staff with shifting power dynamics and the sense that patient-centered approaches might overshadow staff safety and concerns. Crucially, senior leaders and frontline staff framed each other’s resistance differently: staff viewed leaders’ reluctance as retaining power for its own sake, perhaps signaling ongoing mistrust, whereas leaders perceived staff reluctance as holding onto power for safety’s sake. Nevertheless, these moments of ambivalence did not eclipse a sense of possibility. Most participants emphasized that, despite these barriers, forensic POR presents meaningful opportunities to improve the practice and relevance of research. We describe these below.

### 3.3 Theme 3: opportunities

This theme captures participants’ views of forensic POR as an opportunity to foster authentic patient engagement, support patients on an individual level with vocational training opportunities and a sense of purpose and potentially inform organizational and system-level changes.

Both frontline staff and leaders described numerous opportunities for forensic POR to generate constructive and positive outcomes. Participants unanimously viewed forensic POR as a means of supporting that could drive systemic improvement (B, SHL) and “go a bit deeper on these issues” (C, SHL). Staff referred to forensic POR as “a wonderful opportunity” (R, CL) and “an opportunity for patients to have input” (O, CL).

Leaders tended to adopt a system perspective, emphasizing opportunities to achieve change across local, provincial, national, and even international levels. Their comments point to the potential far-reaching implications of forensic POR extending beyond forensic mental health care to partnerships with other organizations, service providers, and hospitals (B, SHL; C, SHL; E, SHL; K, SHL). One clinical leader highlighted the potential for patient perspectives to resonate beyond individual units, suggesting that forensic POR could “[m]ake some feel empowered to know that their words are actually going to be shared with other professionals across the field, possibly internationally” (A, CLL).

Two leaders also noted that forensic POR challenges researchers to move beyond familiar processes, such as relying on patient/family councils, to make relational inroads with patients themselves. This deliberate step into the “uncomfortable” (C, SHL) was described as an opportunity to improve relationships between patients and researchers (H, SHL). We detail a few subthemes about further opportunities below.

#### 3.3.1 Subtheme 3.1: markers of success

Several participants described what indicators of success for forensic POR might look like. These included patients telling researchers they are doing a good job and recognizing that their voices are accurately reflected in research findings (A, CLL); patients reporting a “sense of fulfilment” (W, CL); and practical signs of progress, such as patients “making their boards” (R, CL). Increased patient engagement, reflected in the breadth of representation (V, CL), and high-quality research findings (A, CLL) were also noted as important markers. Participants emphasized knowledge dissemination activities such as “circling results” back to patients (A, CLL), co-presenting at conferences (C, SHL; F, CLL), and co-producing publications “with patients name associated with it” (E, SHL).

One clinical leader described success as the moment when “the coin flips,” when staff and patients begin approaching researchers with ideas or requests for inquiry, suggesting that POR is becoming embedded in the organizational culture:

I think you will know you’re doing a good job when you have buy-in and people that want to participate … when the coin flips a little bit, and staff are saying, Hey, this is a great idea for a research study, or patients are saying, Hey, I’m struggling with this, I would like to … explore this … When we have that coin flip on that engagement side, that’s when we know we’re successful. (H, CLL).

Leaders tended to emphasize organizational and system-level indicators: culture change, diffusion of innovations across sites, and building an evidence (J, SHL) base that could “springboard” successive studies. Staff, conversely, were more likely to identify unit-level outcomes, such as relational improvements, fewer conflicts, and patients’ own reports that care and daily life had improved.

#### 3.3.2 Subtheme 3.2: gaining new skills, keeping busy, and personal benefits

Sustained engagement in research through ongoing involvement in research groups (S, CL) was viewed positively. Participants noted how participation in forensic POR can bolster patients’ vocational best interests: building resumes (S, CL); earning “another certificate or a checkmark on their board report saying I’m a good citizen” (O, CL); creating “something to show to make their effort matter” (U, CL); and documenting productive occupation of time for annual Review Board hearings (F, CLL; H, SHL). These activities were framed as meaningful ways to structure time, foster purpose, and counter the monotony of secure care, which could benefit long-stay patients in particular. As one frontline clinician articulated, benefits of participating in POR could translate into positive relational outcomes on the units:

I think that we would see more patients leave the [hospital]. I think that we would see happier patients. I think that they would have better days. And then they can actually work towards a goal that they’re wanting to work towards, and not necessarily one that we’ve set out for them. I just think it would be great for [seeing] patients who are presenting as more engaged than normal … I think that would probably be a telltale sign that things are improving. [R, CL].

Frontline staff more than leaders also emphasized personal and measurable gains for patient participants (e.g., confidence, fulfillment) and, in some cases, for frontline staff themselves, yet simultaneously worried about balancing them with existing demands and obligations (R, CL; W, CL). Frontline clinicians highlighted relational upsides, such as enhanced therapeutic rapport and patient self-esteem (F, CLL; V, CL; W, CL).

#### 3.3.3 Subtheme 3.3: shiny new object

Healthcare leaders noted the newness of forensic POR and the potential for early adopters to put a positive stamp on this form of research in forensic settings (M, SHL; N, CLL), recently described as a “shiny new object” (40). This positive gloss, they suggested, may attract successive researchers and inspire ongoing projects. Participants also remarked that early successes – “one tiny win … [a] golden nugget … your starting block for your storytelling to change the culture” (I, SHL) – could help build an evidence base that propels future POR studies across similar forensic sites or among similarly situated groups. Several leaders posited that the successful implementation of forensic POR could “have utility beyond [the hospital]” (C, SHL) and be replicated or adapted to other forensic sites and programs (B, SHL; E, SHL). Staff did not typically frame forensic POR in these reputational or innovation-diffusion terms; instead, they anchored their reflections in how any “new” initiative would intersect with existing pressures and day-to-day obligations.

## 4 Discussion

Cumulatively, our findings suggest that mental health care providers and leaders are generally supportive of forensic POR and understand the nuances of individual and structural barriers to participatory research methods in secure environments. However, positive overtures have not yet cohered into a homogeneous genre due to recurrent concerns, primarily raised by frontline staff, about structural and organizational constraints, engagement/motivation, and epistemic questions. Leaders more frequently demonstrated a social justice orientation and a lexicon consistent with Critical Disability Studies and Mad Studies, signaling that they recognize systemic inequities that traditional mental health research practices can reproduce. In contrast, frontline staff often described similar dynamics without naming them in these terms, suggesting a gap in critical vocabulary rather than in everyday observation.

Leaders tended to conceptualize forensic POR as a way to redistribute power to patients. Some staff, however, framed power as something liable to be misused (e.g., worries about patients “showboating”) and emphasized personal safety if power was equalized. Our interpretation aligns with Frisch et al., who argue that decision−makers have “the ability to create conditions in institutions that encourage or discourage partnered research … [and that] top−down approaches to support partnered work can change institutional culture … align[ing] partnered activities with strategic goals” (41). Godin et al. similarly documented service-user accounts that staff “always stick together” and construct (mis)representations in ways that override patient perspectives, highlighting how institutional processes consolidate provider authority (42). In our study, leaders with greatest decisional power appeared most willing, at least rhetorically, to cede power, while staff with less institutional authority voiced more concrete reservations tied to safety risks and workload demands. These differences in perspective help explain why our three participant groups have different views on interpreting risk and on patient participation in research. Leaders were more familiar with social justice and Mad Studies, likely due to their exposure to policy, administration, and system-level discourse. In our view, this gives them a critical vocabulary for naming systemic inequities that frontline staff perhaps see but do not always name. Frontline staff, on the other hand, had a different but equally important embedded knowledge. Because they are close to patients and the daily workings of the hospital, they know more about what forensic POR would ask of patients and of themselves. Their wariness regarding recruitment, manipulation, and safety may not therefore be overtly stigmatizing. In some cases, it demonstrated an understanding of the systemic and relational intricacies of forensic mental health care, even when it bordered on paternalism. This finding is consistent with research showing that healthcare providers in forensic mental health settings continue to face physical and psychological risks (43), in part due to limited structural support for frontline staff shaped by decades of neoliberal healthcare reform (44). The field is still working to reconcile the structural marginalization of forensic patients with structural constraints that may create challenging conditions for staff and patient safety, although conversations about how to find this balance are becoming more common (45).

Leaders were wary of tokenism, the process of involving patients to satisfy policy expectations or to “tinker at the edges” (46), a concern echoed by Demke et al., who critique forms of forced tokenism that position patients as workers in a “psy−industrial research complex?” (39). Daya et al. similarly caution against single−person “representative” roles and call for authentic, bidirectional engagement: “engaging with only a single consumer or survivor as ‘representative’ is tokenistic and futile” (47). A Mad Studies perspective also warns against ‘strategic essentialism’: when psychiatrized people adopt a simplified ‘lived experience’ identity for strategic access to systems, potentially reifying “normalcy/difference as embodied truths” (48). Staff anticipated that some patients might act strategically when deciding whether to join forensic POR for vocational benefits, Review Board credentials, or leverage among other patients and peers. These concerns point to the importance of transparent role design, inclusion of multiple patients on research teams, and co−produced governance to forestall both tokenism and the incentives that feed it.

Forensic patients often experience “double stigma” for their criminal justice involvement and psychiatric diagnosis, with material effects on housing, legal treatment, and recovery (7). In our findings, however, many instances labelled as stigma, particularly by leaders, reflect a more complex blend of discrimination and sanism. These accounts often moved beyond interpersonal prejudice to recognizing upstream structural violence and structural discrimination that shape who enters the forensic system and under what conditions. To us, this indicates that some participants were naming systemic barriers rather than simply stigma in an individual sense. These findings align with Crocker and Leclair’s recent assertion that advancing forensic mental health research requires a focus on broader structural conditions rather than defaulting to individualized understandings of mental illness that reinforce the pathologization, marginalization, and punishment of forensic patients (45). Sanism, which suffuses “all aspects of the forensic mental health system” (49), more precisely captures this institutionalized dimension of inequity. Following Perlin (2013), sanism can be defined as “an irrational prejudice against people with mental illness, of the same quality and character as other irrational prejudices such as racism, sexism, homophobia, and ethnic bigotry” (50). Naming sanism therefore reveals the institutional and epistemic harms that the concept of stigma alone can obscure. Several staff also privileged healthier or medicated minds as more credible contributors to forensic POR, reinforcing sanist hierarchies in which epistemic worth is ranked by perceived clinical stability.

Resistance to non−expert voices by some participants in our study can be linked to ongoing professional paternalism (51). Paternalism, where clinicians chart trajectories with minimal patient input, remains inhospitable to acting on patient knowledge. For example, some staff questioned whether forensic patients’ accounts are trustworthy and remarked that it is easy for staff to think they know what is best for patients based on established practices. On secure units, a safety−first mandate often narrows participation; as one participant in a UK study highlighted: “On my ward it is safety and security first, involvement and anything else come second to this … Conversely, the safety and security first mantra can be seen to close down talk…” (52). Furimsky et al. likewise warned that providers’ focus on safety can culminate in “restrictive attitudes by staff” (53). Some staff justified silencing based on offence histories, arguing that certain patients forfeited a right to voice, or presumed that a psychiatric diagnosis undermined patients’ articulated experiences (54). Conversely, providers also recognized that patient knowledge carries “clout” and can “enhance the profile of mental health consumer research” (55), and that involving stakeholders can provide a “seal of approval” that “improve[s] [research’s] credibility and worth” (51). Targeted training in social justice competencies could help reconcile organizational commitments to patient−centeredness with frontline concerns about risk and workload demands.

Optimistically, there are glimpses that the psychiatric sciences are opening to participatory inquiry, particularly survivor−led research (56), with patient−led approaches representing the highest “rung” of involvement (57). However, such user−led studies are largely absent in forensic mental health settings, where participatory research is, at present, more feasibly co-led and characterized by bidirectional power from study design through to knowledge translation and dissemination. This positioning, however, while epistemologically pragmatic, sits amid ongoing tensions, often described as a dissonance between clinical research inquiries that foreground positivism and more constructivist and critical perspectives that foreground patient−first subjectivism. A Mad Studies approach to POR might therefore foreground patient voice, maintain a critical orientation to the practices and politics of bio-psychiatric research and knowledge, and challenge epistemological dominance without compromising patient and staff safety. From a Mad Studies perspective, this approach resists “studying down on problemetised bodies” (58), recognizes the “potential in the so−called rants and raves of madpeople” (59), and advances an “ethics of unruliness” (60) to disrupt colonial ways of knowing (61), approaches that we have been unable to locate in forensic mental health research broadly, or in participatory forensic mental health research specifically. Moving closer to these participatory methods might require what Demke et al. term “weaning-off,” meaning partners “without lived experiences must give up privileges and routines if to involve users and survivors in research and clinical practices” (39). We argue that introducing patients to the histories of mad resistance, sanism, and the biopolitics of research has the potential to “madden” forensic POR. Future research might solicit perspectives of scholars and clinicians about how to make forensic POR more compatible with critical methodologies and epistemologies, including hesitations and points of ambivalence, moments of optimism and opportunity, and visions for co-led or fully patient-led forms of inquiry in forensic mental health settings.

Several passages from frontline staff and leaders implied solidarity with prospective patient partners without necessarily naming it. Demke et al. define solidarity as “not excluding … no one at the top and no one at the bottom … no claim to power” (39). Allyship and solidarity resonate with the consumer/survivor research community and suggest that, for partnership to be truly equitable, non-service-user researchers’ allyship should be practiced as a willingness to share interpretive authority and accountability. Building solidarity requires more than procedural inclusion. It asks non−user researchers to cultivate what Chapman calls a “troubled consciousness,” an accountability narrative that confronts the harms of “compulsory sound−mindedness,” “a discursive force … that steers people away from the ethical and political experiences that are fundamental to politicized ethicality and accountability” (62). Relatedly, standpoint theory (63) suggests a “stronger objectivity” when inquiry proceeds from marginalized standpoints, implying that clinicians and hospital leaders who deliberately adopt the standpoint of subjugated patients can arrive at more reflexive and socially situated understandings of forensic POR.

Many staff and leaders were open to change and what forensic POR might bring, especially if it did not compromise safety and fit within daily operations of systems already at maximum capacity. Concerns about time, capacity, cost, and implementation planning recur in the participatory research literature, and especially in forensic participatory research (9, 21, 64, 65). Under-resourcing patient engagement can lead to tokenistic or inconsistent involvement, and the time needed for trust building and authentic engagement frequently conflicts with academic and funding cycles (10, 65). Future implementation should attend to these structural conditions by building in funding and time, rather than assuming that enthusiasm or reputational management alone will carry the work forward. These resourcing and temporal considerations help contextualize participants’ concerns in the study, particularly those raised by frontline staff regarding the feasibility of forensic POR within already constrained systems. A coherent institutional strategy to advance POR, resourcing co−production, scheduling around security routines, and avoiding sanist screening can all help translate forensic POR commitments into practice.

### 4.1 Limitations

This study has several limitations. Participants across the hospital had limited formal training in POR and often conflated POR with broader notions of patient-centred care, which complicated interpretation of their responses. Forensic patients were not involved in data collection or analysis, which we admit limits alignment with POR principles and narrows perspectives to frontline staff and leaders. The study also lacked insight into how participants’ own lived experiences shaped their understandings of POR. Furthermore, the interviewers were all researchers embedded in the hospital’s research institute, and none have any clinical experience, which may have influenced the authenticity of participants’ responses. The study was conducted at a single site, which limits transferability to other forensic mental health settings. A multi-site survey and mixed methods approach covering similar topics would complement these findings and be a suitable direction for future research in Canada and beyond. Finally, participants across the hospital had limited formal training in POR and often conflated POR with broader notions of patient-centred care, which complicated interpretation of their responses. The conceptual boundary between POR as an epistemology and research methodology and patient-centered care as a philosophy and practice in forensic mental health settings warrants ongoing inquiry and clarity.

### 4.2 Future research

Apart from Godin et al. (42), few POR articles in forensic mental health contexts deploy an explicit theoretical base. Future research could: 1) test how Critical Disability or Mad epistemologies shape provider stances by analyzing responses to a case study that uses an explicit social justice theory; 2) examine allyship in forensic research, including whether “mad positive” practices deepen equitable partnership and the productive “hurt” of “giving up power, interpretive authority, control, and certainties” (39); 3) assess how funder requirements (e.g., compulsory patient involvement for CIHR funding) influences provider perspectives; and 4) compare occupational groups (e.g., occupational/recreation therapists, nurses, psychiatrists, psychometrists) to identify discipline specific facilitators and barriers, consistent with Happell et al.’s call for discipline−focused inquiry (15).

### 4.3 Conclusion

The perspectives of healthcare providers, clinical leaders, and senior hospital leaders from a recovery-oriented forensic hospital regarding forensic POR and the inclusion of patient voice in research were analyzed using an inductive thematic analysis approach. The variety of in-facility staff featured in the data ensured that a depth and range of perceptions entered the thematic analysis. Leadership and frontline providers were overall receptive to forensic POR, revealing earnest interest in seeing patient voices featured in research in pursuit of their epistemic emancipation. Few, however, were reluctant to overcome that initial defensive ambivalence, requiring them to relinquish power if they were to become involved in power-equalizing research projects. Our findings redress the informational void that Happell and colleagues (15) noted regarding psychiatric care providers’ stances on service user involvement in research.

## Data Availability

The datasets presented in this article are not readily available to protect the confidentiality of participants. Requests to access the datasets should be directed to: ccanning@waypointcentre.ca.
