# Supplementary material for: Optimism, ambivalence, and opportunities: staff perspectives signaling a critical turn in patient-oriented research in forensic mental health care settings
Source: Front Psychiatry. 2026 May 18;17:1810931. doi: 10.3389/fpsyt.2026.1810931 (PMC13223336; doi:10.3389/fpsyt.2026.1810931)
Supplement: Supplementary file 1 [file SupplementaryFile1.docx]

Supplementary Material

1. **Appendices**

**Appendix A: Participant interview question**

- What do you think of the idea of patient-oriented research in forensic mental health care?
- What do you think are advantages and disadvantages of patient-oriented research in forensic settings, compared to doing research the traditional way (that is, without involving patients)?
- Based on what you know or have experienced, how would you describe the relationship between researchers and frontline staff at the hospital?
- Based on what you know or have experienced, how would you describe the relationship between researchers and forensic patients at the hospital?
- What are some important values or beliefs in forensic mental health care at the hospital?
- How do you think attitudes and beliefs may affect patient-oriented research in forensic mental health care?
- How might hospital policies, strategies, laws, and regulations affect patient-oriented research in forensic mental health care?
- What skills or resources would you or others in your role need to be a part of patient-oriented research?
- What do you think would motivate you or others in your role to be a part of patient-oriented research at the hospital?
- How would we know we are doing a good job of patient-oriented research at the hospital?
- Is there anything else you wanted to add?
